# Medical errors in South Asia

**Published:** 2019-09-10

**Authors:** Sridivya Mukpalkar

**Affiliations:** 1 Editor: Community Eye Health Journal South Asia edition, Hyderabad, India.

**Countries in South Asia deal with medical errors in broadly similar ways, in line with international practice. However, there are differences in approach, and some can do better.**

**Figure:**
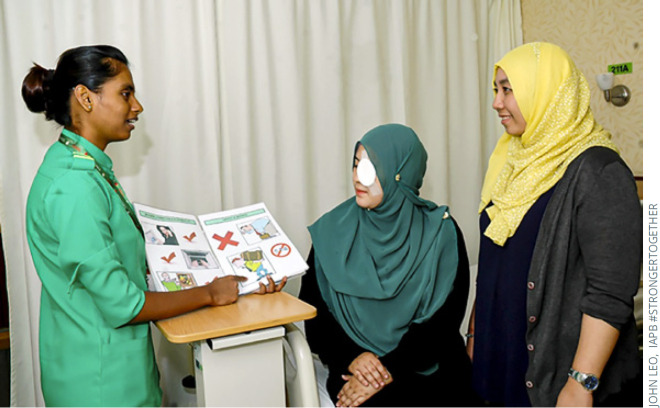
Post-surgery counselling at the Tun Hussein Onn National Eye Hospital. MALAYSIA

As with all types of surgery, eye surgery has inherent risks. It is important that patients understand the prognosis and risks, and that they have realistic expectations about the visual outcome. However, eye surgery doesn't always go as expected. If something has gone wrong, especially if the patient becomes blind, this can be devastating. In these difficult situations, **it is recommended that doctors and/or senior managers step in and communicate directly with the patient** to apologise, say what went wrong and explain what they will do to help the patient.

This approach is not only good for the patient and the family, but also for the hospital and the doctors involved. As a study by Kathleen Mazor et al. notes: “when errors occur, patients and family members are more likely to respond positively when they receive an acknowledgment that what occurred was an error: an explanation of what occurred, an expression of remorse and concern for the patient, and an explicit apology. When the error results in serious and significant harm for the patient, these are even more essential.”^1^ Being honest about errors also makes it possible for medical personnel and institutions to learn and avoid a future occurrence of the same error.

**“It is recommended that doctors and/or senior managers step in and communicate directly with the patient.”**

At Aravind Eye Hospitals in the southern Indian state of Tamil Nadu, if there is a decision during surgery to change the planned surgical technique or intraocular lens, the patient's family is informed, even while surgery is still in progress. A senior doctor or allied ophthalmic professional will break the news to the patient and family members if there has been a surgeon error or a surgical complication. Any error and near miss (a medical error that was noticed and corrected before it caused harm to the patient) is reported immediately on the Incident Reporting System, and detailed notes regarding the error are entered into the patient's medical record. The source of the error is immediately investigated and identified, and every week these errors are reviewed by a committee. Surgical complications are discussed by the relevant surgical team in what is known as the ‘complications meeting.’

In the LV Prasad Eye Institute (LVPEI) network, spread across the states of Andhra Pradesh, Odisha and Telangana in India, doctors and senior managers communicate with the patient when there has been an error or a complication. In case of an error by a surgeon, the operating theatre administrator sends a report that is reviewed by the Quality Department and the director of the Patient Care Department. LV Prasad acknowledges that the impact of a medical error or a complication on a patient is not just short-term, and recommend a psychologist if patients need further support.

In Sri Lanka, many eye hospitals follow a similar approach when handling medical errors. Specialists and senior doctors advise house officers or residents on what should be conveyed to the patients, and what not, in case of a medical error. When the workload is very high for the doctors, the nursing officer or house officer who looks after the patient will speak to them about the error. Although there is no formal protocol for error reporting and managing, informal discussions usually take place among the surgical/clinical team. Counselling is not offered as a routine, but the patient may be referred to low vision and rehabilitation care in appropriate cases. More work needs to be done in Sri Lanka to strengthen the way medical errors are handled, such as telling all patients, including those who are illiterate, when there has been a surgeon or medical error.

In Bangladesh, doctors break the news of an error to the patient and the family. In case of an error, the surgeon, the person in charge of the operating theatre, the medical director and the CEO are informed. An incident form is completed for any error and the senior management takes appropriate action. Depending on the hospital, detailed notes are entered into the patient's medical record by the doctor or senior operating theatre nurse.

In Pakistan, medical errors are handled in a structured way. Patients and managers are informed immediately, and managers will take corrective action and seek to learn lessons from the incident. However, there is room for improvement in all sectors and amongst all stakeholders providing eye care; this has been highlighted by Pakistan's National VISION 2020 Committee and efforts are underway to develop national guidelines.
